# Extended dosing of darbepoetin alfa in peritoneal dialysis patients

**DOI:** 10.1186/1471-2369-12-13

**Published:** 2011-03-24

**Authors:** Mariano Feriani, Johan MJ De Meester, Lawrence P McMahon, Jacques B Rottembourg, Ian Bridges, Mourad Farouk, Wolfgang Pronai

**Affiliations:** 1 Reparto di Nefrologia e Dialisi, Ospedale dell'Angelo, Via Pacagnella, 30174 Mestre, Italy; 2 Department of Nephrology & Dialysis, AZ, Nikolaas, Moerlandstraat 1, 9100 Sint-Niklaas, Belgium; 3 Director, Department of Renal Medicine, Eastern Health, c/- Arnold Street, Box Hill 3128, Victoria, Australia; 4 Dialysis Unit, Centre Suzanne Levy, Paris 75011, France; 5 Biostatistics, Amgen Ltd, 240 Cambridge Science Park, Milton Road, Cambridge, CB4 0WD, UK; 6 Clinical Development, Amgen (Europe) GmbH, Dammstrasse 23, 6300 Zug, Switzerland; 7 Department of Internal Medicine, Hemodialysis, Barmherzige Brüder Eisenstadt, Esterhazystrasse 26, A-7000 Eisenstadt, Austria

## Abstract

**Background:**

Anemia is common among peritoneal dialysis (PD) patients, and most patients require erythropoiesis-stimulating agents (ESA) to maintain their hemoglobin concentrations within current guideline recommendations. Darbepoetin alfa is an ESA with a 3-fold longer half-life and greater in vivo biological activity than recombinant human erythropoietin, allowing less frequent dosing that may simplify anemia management in these patients, providing benefits to patients, care givers and health care providers. Clinical studies have confirmed the efficacy and safety of darbepoetin alfa administered at extended dosing intervals. However, there are limited data on the management of anemia with ESAs in PD patients in routine clinical practice. The aim of this multicenter observational study in European and Australian dialysis patients was to evaluate darbepoetin alfa administered once every 2 weeks (Q2W) in routine clinical practice for 12 months.

**Methods:**

PD patients ≥18 years old and converting to treatment with darbepoetin alfa Q2W were eligible for enrollment regardless of previous or current ESA use. Patients enrolled in the study were treated according to local usual clinical practice. Data were collected up to 6 months prior to and 12 months after conversion to darbepoetin alfa Q2W. The primary endpoint was hemoglobin concentration 12 months after conversion to darbepoetin alfa Q2W.

**Results:**

Of the 741 eligible PD patients (mean age, 61 years; male, 57%), 640 (86%) completed the study. Mean hemoglobin concentration (g/dL) was 11.69 (95% CI, 11.53-11.86) 6 months before the conversion, 12.25 (95% CI, 12.13-12.38) at conversion, and 11.88 (95% CI, 11.74-12.02) 12 months after conversion to darbepoetin alfa Q2W. The weekly equivalent ESA dose (μg/wk) was a geometric mean of 25.24 (95% CI, 23.46-27.15) 6 months before conversion, 20.90 (95% CI, 19.13-22.83) immediately before conversion, 18.89 (95% CI, 18.13-19.68) at conversion and 19.04 (95% CI, 17.69-20.49) 12 months after conversion. Twelve months after conversion, 70% of patients were receiving darbepoetin alfa Q2W and 73% had hemoglobin concentrations >11.0 g/dL.

**Conclusion:**

In this large observational study, PD patients were able to maintain mean hemoglobin concentrations >11.0 g/dL after conversion to extended dosing of darbepoetin alfa Q2W, with no mean dose increase.

## Background

Anemia is a frequent and significant complication of chronic kidney disease (CKD) [1-3]. Current guidelines recommend the use of erythropoiesis-stimulating agents (ESAs) for the treatment of anemia of CKD [4-6]. Anemia management with ESAs has been associated with a reduction in clinical complications due to anemia, and may improve quality of life [5,7,8].

Peritoneal dialysis (PD) accounts for around 18% of renal replacement therapy in Europe, although figures differ substantially between countries [9,10]. Anemia is common among PD patients, and most require ESAs in order to maintain hemoglobin concentrations within guideline recommendations [4,5,11].

ESAs stimulate erythropoiesis through the same mechanism as erythropoietin, the endogenous glycoprotein hormone [3,12]. Darbepoetin alfa (Aranesp^®^, Amgen Inc, Thousand Oaks, CA) has an increased sialic acid-containing carbohydrate content, resulting in an approximately 3-fold longer half-life and greater in vivo biological activity than recombinant human erythropoietin (rHuEPO), consequently darbepoetin alfa can be administered at extended dosing intervals, such as up to once every 2 weeks (Q2W) for dialysis subjects or once a month for non-dialysis subjects (13-15).

Clinical trials have confirmed the efficacy and safety of darbepoetin alfa Q2W in the management of anemia in patients receiving dialysis, including those on PD [13-16]. However, there are few data on the actual management of anemia with ESAs in PD patients in routine clinical practice. To contribute towards establishing more data on the use of a Q2W extended dosing regimen in this setting, ALTERNATE (A Long-term Non-interventional Study to Evaluate the Effectiveness of Aranesp^® ^in Dialysis Patients When Administered Once Every Two Weeks) was undertaken. This long-term observational study followed both hemodialysis and PD patients treated with darbepoetin alfa Q2W. In this report, we describe the PD subgroup.

## Methods

This was an observational, multi-national study carried out at 344 centers in Europe and Australia. Patients ≥18 years of age, undergoing routine dialysis (hemodialysis or PD) and initiating treatment with darbepoetin alfa Q2W were eligible for inclusion. All eligible patients were enrolled, and enrollment continued sequentially until the target number of patients was reached. The final patient completed the study in September 2008. Patients were excluded if they had received darbepoetin alfa in an interventional study within 6 months of converting to darbepoetin alfa Q2W, were receiving other investigational agents, or had clinical evidence of current malignancy with the exception of basal cell or squamous cell carcinoma of the skin and cervical intraepithelial neoplasia.

Data were abstracted from patient charts for up to 6 months prior to and 12 months after conversion to darbepoetin alfa Q2W. Data were abstracted retrospectively for the 6 months prior to conversion, and retrospectively and prospectively for the 12 months after conversion, depending on the date of enrolment in the study. As this was an observational study, there were no pre-specified anemia management guidelines or monitoring, and patients were treated according to the routine clinical practice in the respective treatment center. Where required by local law, the study protocol and informed consent materials were approved by the local Independent Ethics Committee, and the patients provided written informed consent prior to study entry.

### Statistical analysis

For the overall study, a sample size of approximately 6000 patients was calculated as the number of patients needed for the results to have adequate precision for pre-specified patient subgroups with only 5% of the total sample (n = 312). The PD subset accounted for 5.1% of the total study population with over 700 enrolled patients.

In the overall study, the primary endpoint was the hemoglobin concentration 12 months after conversion to darbepoetin alfa Q2W. Secondary endpoints included the proportion of patients within European Best Practice Guidelines treatment targets for hemoglobin (>11 g/dL) [5], ESA utilization and iron usage, hospitalization and darbepoetin alfa-related adverse drug reactions (ADRs) during the 12-month post-conversion period.

Pre-specified analyses were carried out for the PD subgroup. Continuous variables are described using mean, standard deviation (SD), 95% 2-sided confidence intervals (CIs), median, and quartile ranges (Q1, Q3). Categorical variables are summarized as the number and percentage of patients.

Analysis of monthly hemoglobin was based on the value closest to the date of the scheduled time point (eg, month 1, 2, 3) within a ± 2-week window, with the exception of the Q2W conversion time point which included values up to 8 weeks before conversion to ensure the maximum number of patients for evaluation. Intrapatient variability was calculated for each patient as the standard deviation of all monthly hemoglobin results for the period.

ESA doses were expressed as geometric means (log transformation of the data) due to the non-normal distribution of the data. Epoetin doses were converted from IU to μg using European (200:1) or Australian (200:1 SC or 240:1 IV) product label guidelines. The weekly equivalent ESA dose was calculated for individual patients for each month using all available dosing data within that month.

Analyses of endpoints included all eligible patients enrolled in the study and converted to darbepoetin alfa Q2W (full analysis set [FAS]) as observed (no imputation for missing values), regardless of subsequent change in regimen (dose, frequency, or ESA). To assess the effect of patients' withdrawal or missing data on hemoglobin concentration, a FAS last observation carried forward (LOCF) analysis was also undertaken for the primary endpoint. Additional post-hoc analyses were undertaken to assess hemoglobin and dose levels in patients treated with darbepoetin alfa only prior to conversion to darbepoetin alfa Q2W, and whether the inclusion of ESA-naïve subjects affected the mean dose and hemoglobin levels. All statistical analyses were performed with SAS/STAT software (version 8.2, SAS Institute Inc., Cary, NC, USA).

The ALTERNATE database and statistical analyses were managed by the study sponsor.

## Results

A total of 742 PD patients were enrolled and 741 were eligible for the study from 172 centers in 14 European countries (n = 593) and Australia (n = 148). One patient was ineligible as age was not confirmed as ≥ 18 years. Six hundred and forty patients completed the study (86.4%); the main reasons for discontinuation were kidney transplant (n = 39), and death (n = 36) (Table 1).

**Table 1:** Subject disposition

	n (%)
Eligible subjects (full analysis set)	741
Subjects who completed study	640 (86.4)
Subjects who discontinued study	101 (13.6)
Kidney transplant	39 (38.6)^a^
Death	36 (35.6)^a^
No longer treated at clinic	15 (14.9)^a^
Consent withdrawn	1 (1.0)^a^
Other	10 (9.9)^a^

^a^Percentage of all subjects who discontinued the study

Patient demographics and characteristics at conversion are shown in Table 2. Immediately before conversion, most patients were receiving darbepoetin alfa (76.1%), and most had a weekly (QW) ESA regimen (78.4%). In total, 8.9% of patients had not received an ESA in the 6 months prior to conversion to darbepoetin alfa Q2W (including ESA-naïve patients and patients who stopped ESA therapy for 6 months or more).

**Table 2:** Subject demographics and characteristics at conversion to darbepoetin alfa Q2W

	n = 741
Sex, n (%)	
Female	318 (42.9)
Male	421 (56.8)
Not recorded	2 (0.3)
Age (years), mean (SD)	61.2 (15.2)
Age (years), n (%)	
<65	413 (55.7)
≥65	328 (44.3)
Primary cause of CKD, n (%)	
Diabetes mellitus	140 (18.9)
Glomerulonephritis	139 (18.8)
Hypertension	137 (18.5)
Unknown etiology	108 (14.6)
Polycystic kidney/hereditary disease	67 (9.0)
Interstitial nephropathy/obstructive nephropathy	63 (8.5)
Tumors	6 (0.8)
Other	81 (10.9)
Duration of dialysis (years), mean (SD)	3.1 (3.6)
Duration of dialysis (years), n (%)	
<2 years	231 (31.2)
2-5 years	429 (57.9)
>5 years	81 (10.9)
History of cardiovascular disease, n (%)	379 (51.1)
History of diabetes, n (%)	210 (28.3)
Type 1	30 (4.0)
Type 2	173 (23.3)
Diabetic, type not recorded	7 (0.9)
Duration of ESA use^a ^(years), mean (SD)	2.8 (2.0)
Hb concentration at conversion, g/dL, n (%)	
≤11	167 (22.5)
>11-13	307 (41.4)
>13	228 (30.8)
Not recorded	39 (5.3)
ESA treatment prior to conversion, n (%)	
Darbepoetin alfa	564 (76.1)
Epoetin alfa	19 (2.6)
Epoetin beta	92 (12.4)
ESA naïve^b^	66 (8.9)
ESA frequency prior to conversion^c^, n (%)	
TIW or BIW	20 (3.0)
QW	529 (78.4)
Other	124 (18.4)
Not recorded	2 (0.3)
ESA route prior to conversion^c^, n (%)	
IV	41 (6.1)
SC	634 (93.9)
Darbepoetin alfa route at conversion, n (%)	
IV	24 (3.2)
SC	717 (96.8)
Darbepoetin alfa weekly dose at conversion, μg/wk	
Geometric mean (95% CI)	18.89 (18.13-19.68)
Iron status, n (%)	
Serum ferritin >100 μg/L and either TSAT >20% or hypochromic red cells <10%	255 (34.4)
Serum ferritin ≤100 μg/L or both TSAT ≤20% and hypochromic red cells ≥10%	161 (21.7)
Not recorded	325 (43.9)

^a^n = 718; ^b^received no ESA in the 6 months prior to conversion; ^c^percentages calculated excluding ESA-naïve patients;

Abbreviations: BIW, twice a week; CI, confidence interval; CKD, chronic kidney disease; ESA, erythropoiesis-stimulating agent; Hb, hemoglobin; IV, intravenous; QW, once a week; Q2W, every two weeks; SC, subcutaneous; SD, standard deviation; TIW, three times a week; TSAT, transferrin saturation

### Hemoglobin and ESA dose

**Figure 1:**
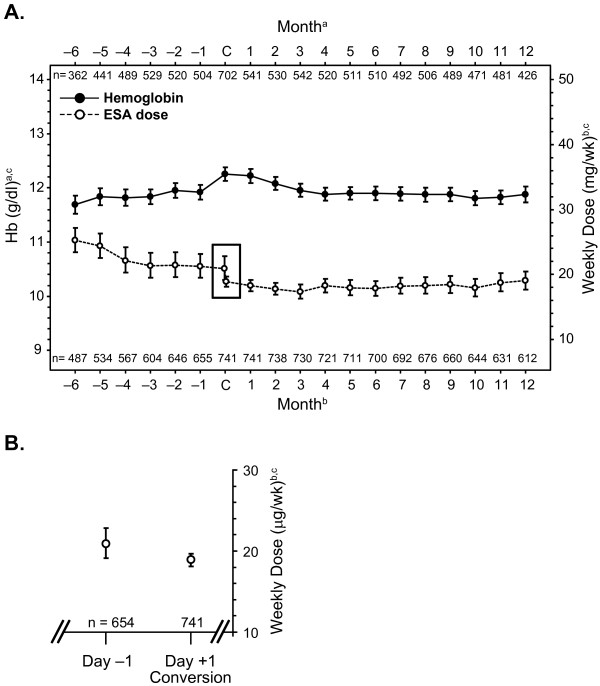
**(A) Mean hemoglobin (g/dL) and calculated geometric mean weekly erythropoiesis-stimulating agent dose (μg/wk; ± 95% CI) by month in the FAS; (B) Weekly dose day prior to conversion and first dose of darbepoetin alfa**. The mean hemoglobin concentration 6 months prior to conversion to darbepoetin Q2W was 11.69 (95% CI, 11.53-11.86) g/dL, trending upwards to 12.25 (95% CI, 12.13-12.38) g/dL at conversion. At month 12 after conversion, the mean hemoglobin concentration was 11.88 (95% CI, 11.74-12.02) g/dL. The calculated weekly dose of ESA decreased from a geometric mean of 25.24 (95% CI, 23.46-27.15) μg/wk 6 months prior to conversion to 20.90 (95% CI, 19.13-22.83) μg/wk the day before conversion (Figure 1B). At conversion, the calculated first dose of darbepoetin alfa was a geometric mean of 18.89 (95% CI, 18.13-19.68) μg/wk (Figure 1B), and 19.04 [95% CI, 17.69-20.49] μg/wk at month 12. ^a^Hemoglobin each month was defined as the single closest value in a ± 2 week analysis window; at conversion, it was the single closest value in a -8 week analysis window; ^b^Weekly dose was the total dose received each month (30 days) converted to a weekly equivalent; ^c^No imputation for missing values; Day -1 was the dose of the regimen the day before conversion (converted to a weekly equivalent); Day +1 (conversion) was the first dose of darbepoetin alfa Q2W (converted to a weekly equivalent) Abbreviations: wk, week; CI, confidence interval; C, at conversion to darbepoetin alfa every 2 weeks; Hb, hemoglobin FAS, full analysis set; Q2W, every 2 weeks;. darbepoetin alfa every 2 weeks; ESA, erythropoietin stimulating agent; FAS, full analysis set; Hb, hemoglobin.

The mean hemoglobin concentration 6 months prior to conversion to darbepoetin Q2W was 11.69 (95% CI, 11.53-11.86) g/dL, trending upwards to 12.25 (95% CI, 12.13-12.38) g/dL at conversion (Figure 1) in the FAS. At month 12 after conversion, the mean hemoglobin concentration was 11.88 (95% CI, 11.74-12.02) g/dL (Figure 1) in the FAS and 11.83 (95% CI, 11.72-11.94) g/dL in the LOCF analysis.

**Figure 2:**
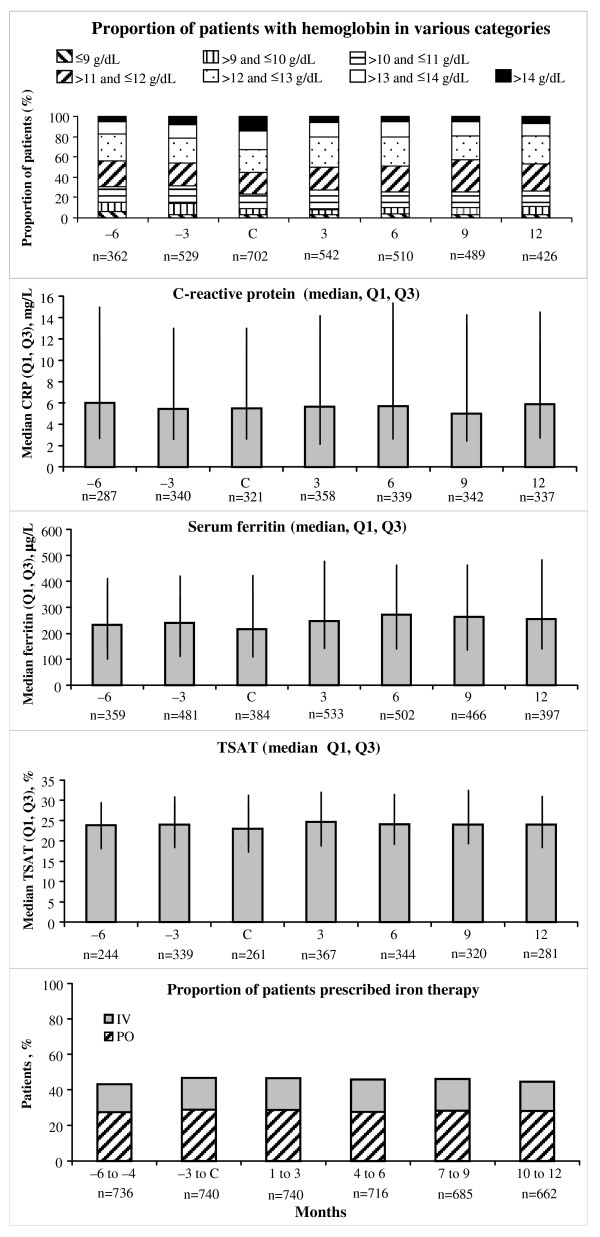
**Proportion of patients with hemoglobin concentration in various categories, C-reactive protein (CRP) levels, iron stores and usage before and after conversion to darbepoetin alfa Q2W**. Most patients (73.0%) achieved a hemoglobin concentration >11 g/dL at month 12, which was similar to the proportion of patients 6 months prior to conversion (69.3%) and at conversion (76.2%). The proportion of patients achieving a hemoglobin concentration >11 and ≤13 g/dL was 52.5% 6 months prior to conversion, 43.7% at conversion, and 54.0% at month 12. CRP and iron levels were stable over the study. Between 43.2% and 46.6% of patients were on iron therapy in any 3-month period during the study. Of those patients on iron therapy, about 60% of patients were receiving oral iron therapy. Abbreviations: C, at conversion to darbepoetin alfa every 2 weeks; CRP, C-reactive protein; PO, oral; IV, intravenous; Q1-Q3, quartile ranges; TSAT, transferrin saturation.

Most patients (73.0%) achieved a hemoglobin concentration >11 g/dL at month 12 (Figure 2), which was similar to the proportion of patients 6 months prior to conversion (69.3%) and at conversion (76.2%). The proportion of patients achieving a hemoglobin concentration >11 and ≤13 g/dL was 52.5% 6 months prior to conversion, 43.7% at conversion, and 54.0% at month 12.

Mean intrapatient variability in hemoglobin was reduced following conversion to darbepoetin alfa Q2W, from 0.79 g/dL and 0.90 g/dL 6 to 4 months and 1 to 3 months prior to conversion, respectively, to 0.71 g/dL, 0.65 g/dL, 0.70 g/dL, and 0.65 g/dL in the 3-month periods from 1 to 12 months after conversion.

The calculated weekly dose of ESA decreased from a geometric mean of 25.24 (95% CI, 23.46-27.15) μg/wk 6 months prior to conversion to 20.90 (95% CI, 19.13-22.83) μg/wk the day before conversion. At conversion, the calculated first dose of darbepoetin alfa was a geometric mean of 18.89 (95% CI, 18.13-19.68) μg/wk, and 19.04 [95% CI, 17.69-20.49] μg/wk at month 12 (Figure 1). Similar results were observed when only subjects treated with darbepoetin alfa prior to conversion (n = 564) were analyzed, or when ESA-naïve patients (n = 66) were excluded from the analyses [data not shown].

ESA dose remained unchanged in 65.7% and 63.8% of patients in months 1 to 6, and 7 to 12 after conversion, respectively, compared with 71.7% in the 6 months prior to conversion to darbepoetin alfa Q2W. ESA dosing frequency remained unchanged in 79.8% and 74.5% of patients in months 1 to 6, and 7 to 12 after conversion, respectively, compared with 82.8% in the 6 months prior to conversion. Of those patients who had changes in dose and/or frequency, most patients (88%) had 1 or 2 changes. At month 12, 70.0% of patients were receiving darbepoetin alfa Q2W and 5.5% of patients were receiving darbepoetin alfa once a month (Table 3).

**Table 3:** Erythropoiesis-stimulating agent type and frequency at month 12 for peritoneal dialysis patients who initiated darbepoetin alfa Q2W treatment

Frequency	ESA Type (n = 616)		
	**Darbepoetin alfa n (%)**	**Epoetin alfa n (%)**	**Epoetin beta n (%)**

Total	594 (96.4%)	13 (2.1%)	9 (1.5%)
TIW/BIW	5 (0.8%)	5 (0.8%)	2 (0.3%)
QW	94 (15.3%)	3 (0.5%)	4 (0.6%)
Q2W	431 (70.0%)	--	--
QM	34 (5.5%)	--	--
Other	30 (4.9%)	5 (0.8%)	3 (0.5%)

Abbreviations: BIW, twice a week; QW, once weekly; Q2W, once every other week; QM, once monthly; TIW, three times a week

### Laboratory values

C-reactive protein (CRP) and iron levels were stable over the study (Figure 2). In patients whose hemoglobin levels were >11 g/dL for at least 60% of the time following conversion, median (Q1, Q3) CRP levels were lower than in patients with these hemoglobin levels for < 60% of the time (6.0 [2.9, 14.0] vs. 9.0 [3.5, 21.4] mg/L).

Between 43.2% and 46.6% of patients were on iron therapy in any 3-month period during the study (Figure 2). Of those patients on iron therapy, about 60% of patients were receiving oral iron therapy.

### Hospitalizations

**Figure 3:**
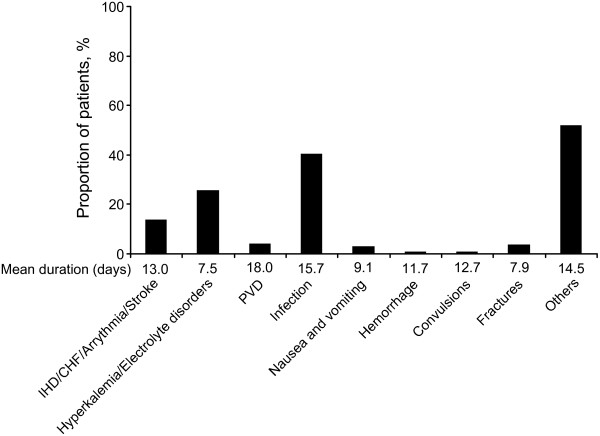
Primary reasons for hospitalization during 12 months after conversion to darbepoetin alfa Q2W*. The primary reasons for hospitalization included infection (40.3%), and hyperkalemia/electrolyte disorders (25.7%), although most reasons for hospitalization were listed as 'other' (51.7%) *Calculated from the patients' who were hospitalized, the percentage who had at least one hospitalization for each reason. Note that more than one cause of hospitalization could be recorded for the same patient. Abbreviations: IHD, ischemic heart disease; CHF, chronic heart failure; PVD, peripheral vascular disease; Q2W, every 2 weeks.

During the 12 months after conversion to darbepoetin alfa Q2W, 52.0% of patients were hospitalized with a mean duration of 18.8 days (median 14.0 days). The primary reasons for hospitalization included infection (40.3%), and hyperkalemia/electrolyte disorders (25.7%), although most reasons for hospitalization were listed as 'other' (51.7%) (Figure 3).

### Safety

The ADR rate was low during the 12 months after conversion to darbepoetin alfa Q2W, with no unexpected events emerging. A total of 6 ADRs were reported in 4 patients (0.5%). These included myalgia (n = 2), hypertension (n = 1), injection site pain (n = 1), muscular weakness (n = 1), and rash (n = 1). A total of 36 patients (4.9%) were withdrawn from the study due to death, but these deaths were considered unrelated to darbepoetin alfa by the investigators.

## Discussion

Currently, ALTERNATE is the largest observational study of ESA use in dialysis patients in Europe and Australia, and includes a relatively large number of PD patients. The results of this analysis in PD patients indicate that despite reducing the frequency of administration, patients converted to darbepoetin alfa Q2W had a mean hemoglobin concentration >11.0 g/dL 12 months after conversion, without requiring an increase in mean dose. Moreover, 70% of patients were receiving darbepoetin alfa Q2W at month 12, indicating that most PD patients were successfully maintained on extended dosing frequency.

In order to recruit a large, diverse population, our study had few eligibility criteria. This study included patients who were ESA naïve, and patients who converted from other ESAs and dosing regimens. Indeed, the characteristics and hemoglobin concentrations at conversion were similar to the Netherlands Cooperative Study on the Adequacy of Dialysis, which included 480 PD patients [17]. This suggests that our patient population is typical of European PD patients, and the treatment practices observed are representative of the PD population. Hospitalizations were frequently reported in our study, which is typical of a dialysis population, and the types of ADRs related to darbepoetin alfa were consistent with those reported in controlled trials of dialysis patients receiving darbepoetin alfa [13,14,16]. Our study reports that hemoglobin concentrations can be maintained >11 g/dL without an increase in mean dose after conversion to extended (Q2W) darbepoetin alfa dosing in clinical practice. These results are consistent with randomized controlled trials of extended Q2W dosing of darbepoetin alfa in dialysis patients (hemodialysis and PD patients) [5,16,18], and several smaller studies in PD patients [14,15,19]. Data from a pooled subanalysis of eight trials also reported that PD patients can be switched from epoetin alfa or beta to darbepoetin alfa Q2W regimen without a dose increase and with maintenance of hemoglobin concentrations [20].

Iron stores were within the European guideline targets in most patients and up to 47% of patients in any 3-month period were prescribed iron supplementation in this study [5]. Iron deficiency was reported in 39% of patients with sufficient data to assess iron deficiency at conversion. This is of concern as iron deficiency can inhibit the response to ESAs, and regular assessment is recommended by European guidelines [5,11].

An upwards trend in hemoglobin concentration was observed prior to conversion to darbepoetin alfa Q2W. This may provide insight into the reasons for conversion to an extended dosing regimen, with physicians less likely to change an existing treatment regimen in patients with well controlled hemoglobin, than in patients who have rising hemoglobin. In addition, hemoglobin was not stable in many patients in the 3 months prior to conversion, as reflected in the intrapatient variation, which may have influenced the decision to switch. The data also indicate a reduction in dose at conversion, which could suggest that these patients do not receive the equivalent dosage when converted to an extended dosing regimen, resulting in a lower hemoglobin after conversion.

Extended dosing may be advantageous in the management of anemia in PD patients as it reduces the frequency of ESA administration and may enable alignment of ESA administration with routine clinic visits, especially since PD patients as a group are not seen as often by health care providers as hemodialysis patients [15]. This could reduce both the number of visits and injections required by a patient each month, and decrease the potential for injection site pain, which may increase patient satisfaction and improve adherence to treatment [21-23]. ESA management is also labor intensive and time consuming for health care providers, requiring frequent hemoglobin monitoring to ensure that guideline targets are maintained, dose changes for hemoglobin variation above guideline ranges, and depending on the ESA half-life, administration up to three times a week to maintain appropriate hemoglobin concentrations [23]. Thus, extended dosing may simplify anemia management for patients, care givers, and health care providers. Our study indicates that PD patients can be successfully converted to an extended dosing regimen, with 70% of patients receiving darbepoetin alfa Q2W at month 12.

There are a number of potential limitations to this study. As this was an observational study, there were no anemia management guidelines or monitoring, therefore there may have been differences in hemoglobin targets and reimbursement for the management of renal anemia in individual centers and countries. Additionally, there was no control group or randomization, which limits the statistical assessment of the data. There may have been selection bias for patients included in our study, however, the eligibility criteria was aimed at recruiting a wide, diverse population, and the study protocol required patient enrollment to be sequential, therefore centers could not select specific patients. As noted above, the results of this study indicate that the decision to convert to darbepoetin alfa Q2W may have been biased toward patients with increasing hemoglobin prior to conversion. This may reflect the underlying reasons for conversion to an extended dosing regimen, rather than an inherent bias. ESA-naive patients were also included in the analysis, but post-hoc analysis indicated that they did not impact the results. As observational studies may be limited by missing data points, we included a sensitivity analysis in which the data were analyzed by applying LOCF imputation to evaluate the effects of missing values and changes in ESA treatment after conversion to darbepoetin alfa Q2W; results were comparable.

In March 2008, during the ALTERNATE study, the label changed for darbepoetin alfa in Europe. The recommended hemoglobin target was changed from >11.0 g/dL (with an individualised upper limit of <14.0 g/dL) to a target of 10.0-12.0 g/dL for all patients. Therefore the data for patients achieving the previously recommended hemoglobin target of >11.0 g/dL are no longer exactly aligned with current targets, and these results may give the appearance that patients were not achieving target ranges.

## Conclusions

Results from this observational study with darbepoetin alfa indicate that PD patients can successfully maintain hemoglobin concentrations within guideline targets in a clinical practice setting without an increase in the mean dosage using a darbepoetin alfa Q2W regimen, and provides insights into the treatment practices of PD patients receiving ESAs.

## Competing interests

This study was funded and sponsored by Amgen (Europe) GmbH. Drs Mariano Feriani, Johan MJ De Meester, Lawrence P. McMahon, Wolfgang Pronai and Jacques B Rottembourg are members of the ALTERNATE Steering Committee. Dr Johan MJ De Meester has received consultancy fees and speaker fees, and Dr Lawrence P McMahon has received consultancy fees from Amgen. Ian Bridges and Mourad Farouk are employees of Amgen (Europe) GmbH.

## Authors' contributions

MF has contributed to the data analysis, drafting/revising the content of the manuscript, and read and approved the final manuscript; JDM has contributed to the data acquisition/analysis, drafting/revising the content of the manuscript, and read and approved the final manuscript.

LM has contributed to the data acquisition/analysis, drafting/revising the content of the manuscript, and read and approved the final manuscript; JR has contributed to the data acquisition/analysis, drafting/revising the content of the manuscript, and read and approved the final manuscript; IB has contributed to the data acquisition/analysis, drafting/revising the content of the manuscript, and read and approved the final manuscript; MF has contributed to the data acquisition/analysis, drafting/revising the content of the manuscript, and read and approved the final manuscript; WP has contributed to the data acquisition/analysis, drafting/revising the content of the manuscript, and read and approved the final manuscript.

## Pre-publication history

The pre-publication history for this paper can be accessed here:

http://www.biomedcentral.com/1471-2369/12/13/prepub
